# Supplementary material for: A Five-Year Malaria Prevalence/Frequency in Makenene in a Forest–Savannah Transition Ecozone of Central Cameroon: The Results of a Retrospective Study
Source: Trop Med Infect Dis. 2024 Oct 7;9(10):231. doi: 10.3390/tropicalmed9100231 (PMC11511469; doi:10.3390/tropicalmed9100231)
Supplement: Supplementary file 1 [file tropicalmed-09-00231-s001.zip › tropicalmed-3175735 - suppl.pdf]

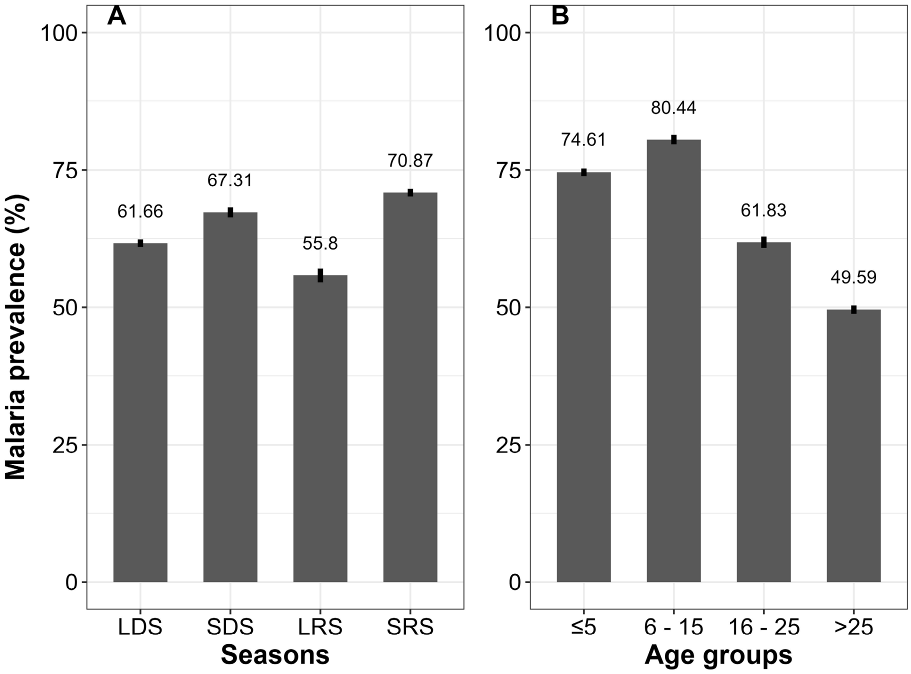

Figure S1. Variation of cumulative malaria prevalence during the study period (**A**) by seasons of the year and (**B**) ages of the participants.
